# Oral manifestation as the only sign of Langerhans cell histiocytosis: A case report

**DOI:** 10.1002/ccr3.8410

**Published:** 2024-01-10

**Authors:** Bahareh Nazemisalman, Mobina Sadat Zarabadi

**Affiliations:** ^1^ Department of Pediatric Dentistry, School of Dentistry Zanjan University of Medical Sciences Zanjan Iran

**Keywords:** Langerhans cell histiocytosis, oral manifestation, pediatric dentistry

## Abstract

**Key Clinical Message:**

The manifestations of Langerhans cell histiocytosis can be limited in the oral cavity, including gingival recession, tooth mobility, and bone destruction. Dentists play a crucial role in the early detection and management of these oral symptoms, which can significantly impact the overall prognosis and quality of life for patients with this serious disease.

**Abstract:**

The hyperplastic activity of bone marrow can lead to excessive histocyte proliferation, called Langerhans cell histiocytosis (LCH). Based on the accumulation location, it has various organ involvement, including skin, bone, and so forth. Oral manifestations such as tooth involvement, bone lesions, mucosal ulcers, and periodontal problems can occur, and they might be the only manifestation of LCH. A subtle diagnosis is crucial and can be started with dental examinations. A 5‐year‐old girl was examined with the chief complaint of gingival recession with no pain, caries, or calculus. The clinical and radiographical assessment led to further immunohistochemical (IHC) evaluations. The diagnosis was LCH based on S‐100 and cluster of differentiation 1a (CD1a) markers. LCH can involve different cells and tissues locally or generally. The early detection and treatment of LCH increase children's survival rate and quality of future life. Therefore, an accurate diagnosis is important among dentists. They must consider that abnormal mobility, gingival, or periodontal problems with no logical cause might warn about a latent problem. Sometimes extraction of loose teeth is not the end of treatment; it is the start of neglecting a serious disease.

## 1 INTRODUCTION

Langerhans cell histiocytosis (LCH), a rare disease of unknown pathogenesis, is characterized by the hyperplastic activity of bone marrow and subsequent accumulation of histiocytic cells in bone, skin, or mucosal tissues.^1^ The prevalence of LCH varies from 0.5 to 5.4 cases per million people annually, with less than 10% involving the jaw.^2^ The etiology of LCH is unclear, however, possible causes, such as neonatal infection or thyroid disease have been suggested.^3^ A subtle diagnosis requires clinical, histopathological, and immunohistochemical (IHC) examination. However, for a definite clinical decision, positive cluster of differentiation 1a (CD1a) and S‐100 markers are essential.^4^ LCH presents with a wide range of clinical manifestations, from single self‐limiting lesions to multisystem conditions that might involve risk organs, such as the liver, spleen, or bone marrow, and treatments vary from local debridement to intensive therapy.^5^, ^6^, ^7^ Histocyte infiltration in the maxilla and mandible can lead to mucosal, periodontal, or bone lesions.^1^ Oral manifestations of LCH include periodontal lesions, hypermobility, early loss of teeth, and inflammation/bleeding/recession in the gingiva.^8^ Differential diagnoses for LCH based on histocyte infiltration site, include pubertal periodontitis, hypophosphatasia, leukemia, and so forth.^3^ It should be noted that oral manifestations might be the first and only affected area, making an accurate diagnosis essential, and dental care providers must be aware of the oral signs of LCH.^1^

This article provides information on possible signs and symptoms, radiographical, histopathological, and IHC assessments based on a case presentation of a 5‐year‐old girl with oral LCH and hypodontia.

## 2 CASE DESCRIPTION

A 5‐year‐old girl was examined, complaining of gingival recession and floating primary molar teeth without pain, or inflammation. Parents were asked about the previous episodes of pain, swelling, and other oral manifestations and they reported none. She was initially seen by a general dentist and then referred to a pediatric dentist for further examinations. A complete medical and psychosocial history were obtained from her family members; there were no record of cancer, genetic diseases, or psychological problems. The patient was born in the third pregnancy with average weight and height, and had no severe systemic illnesses since then.

**FIGURE 1:**
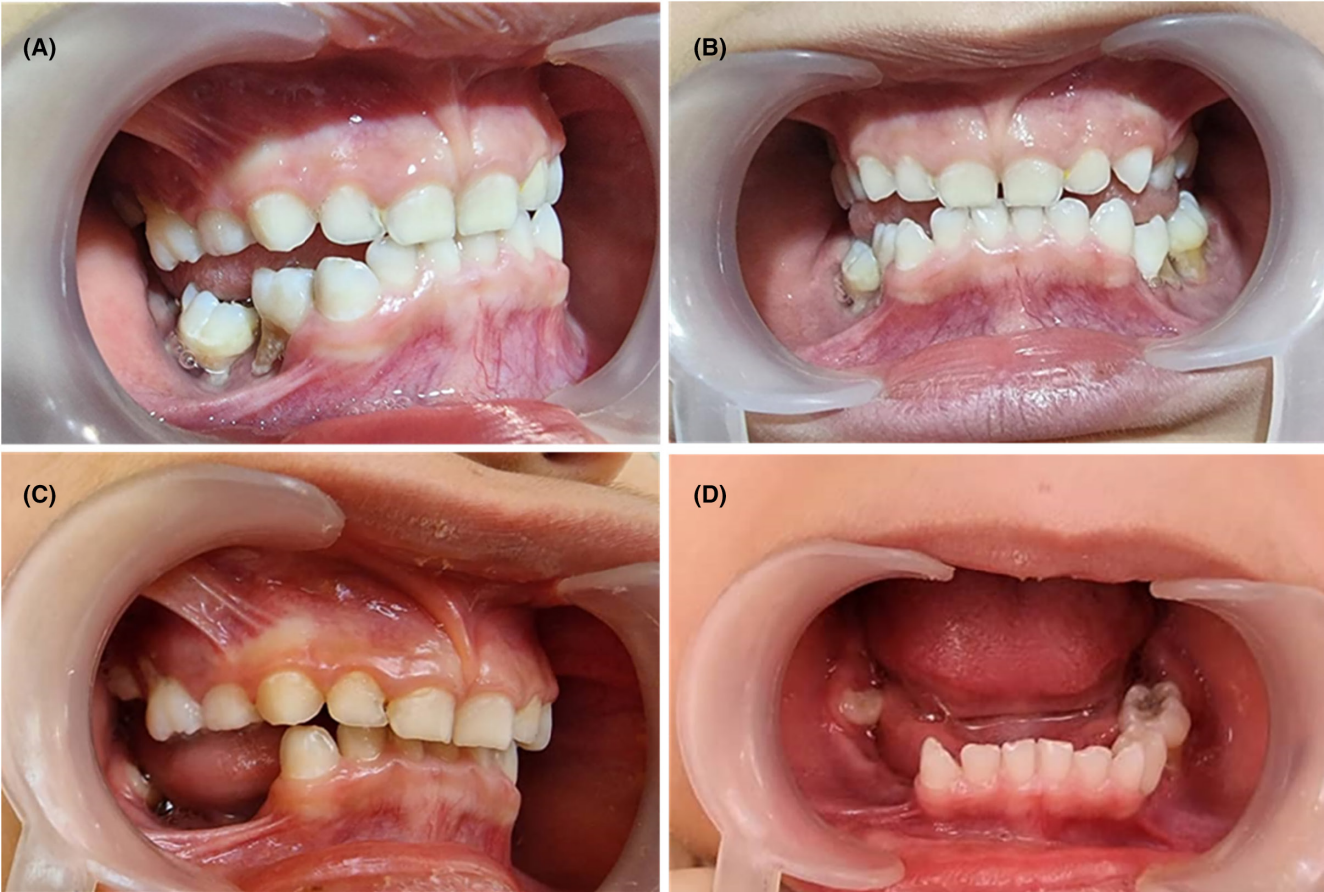
(A, B) Clinical findings showed bone loss with exposed roots and attachment loss in primary mandibular and maxillary molars. (A) lateral view; (B) frontal view (C, D) The follow‐up session after biopsy extraction showed normal socket healing without infection or bone necrosis. (A) Lateral view; (B) frontal view.

Clinical intraoral examination showed gingival recession and attachment loss with no calculus, or inflammation. There was a periodontal pocket with necrotic interdental papilla, visible furcation, and exposed roots in the first and second primary molars on both sides of the mandible and maxilla (Figure 1). Neither pain, halitosis, extensive caries, calculus, bleeding on probing, nor skin involvement was seen. The grade of hypermobility was Grade 2. Ophthalmological, dermatological, cardiac, neurological, and endocrinal evaluations were done with no specific findings.

**FIGURE 2:**
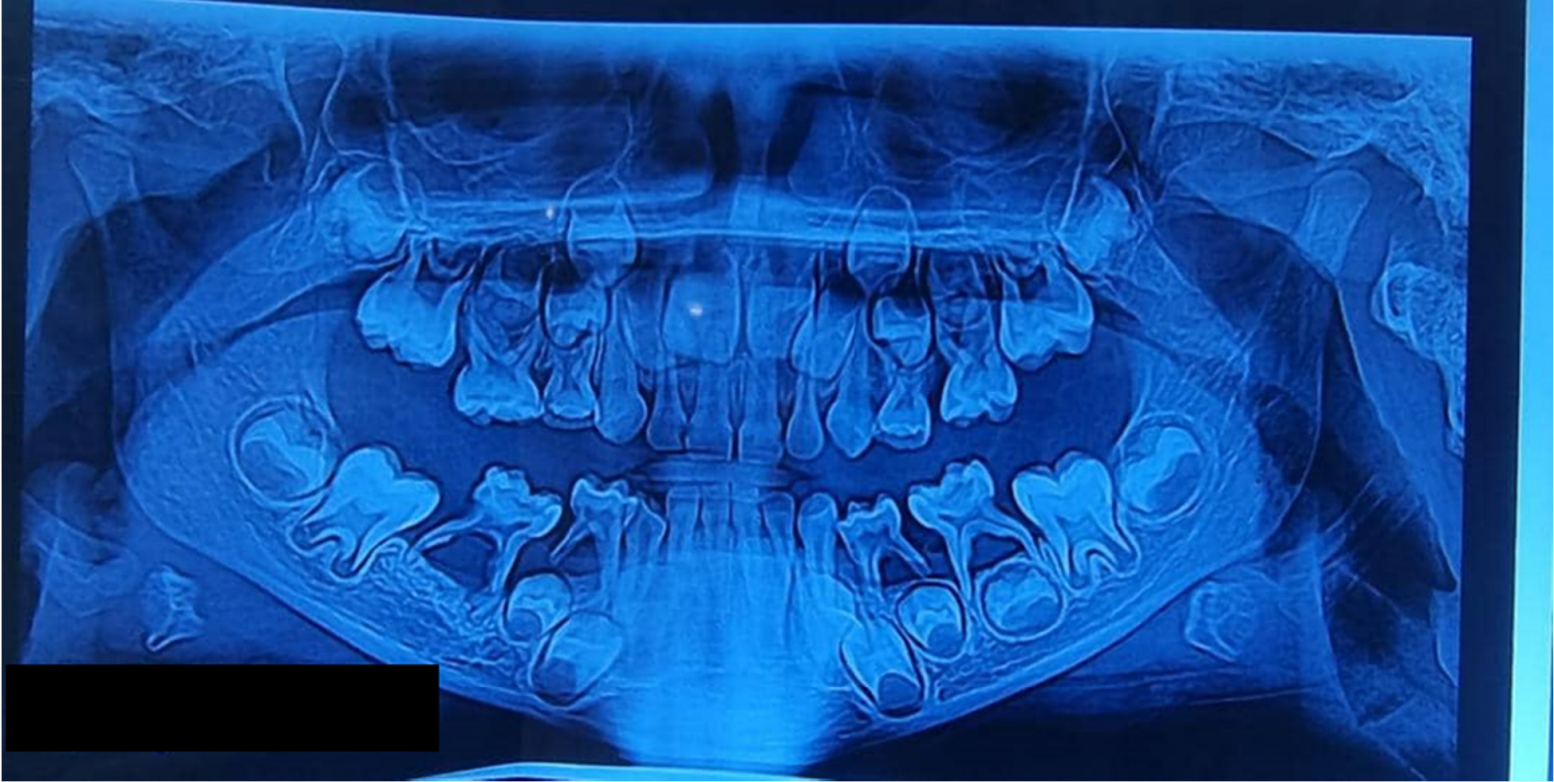
Panoramic radiography. Periodontal destruction and bone loss led to the “floating teeth” view. Notice to the right mandibular area that, the second premolar bud is absent.

After observing idiopathic attachment loss without inflammation or calculus, consultations were taken with a periodontologist and pediatric dentist, and then a blood test and panoramic radiography were ordered. Hematology and blood test biochemistry demonstrated normal blood cell count, alkaline phosphatase, fast blood sugar (FBS), thyroid hormones, calcium, and phosphorus. Panoramic radiography demonstrated radiolucency around mandibular primary molars. Teeth were floated due to destructed lamina dura and bone loss in a scooped‐out shape. The second premolar bud on the right side was absent, and hypodontia was presented (Figure 2).

Attachment loss brings the diagnosis into periodontists, and due to the patient's age, pre‐pubertal periodontists were among the differential diagnoses. No inflammation, edematous gum, or bleeding on probing were seen. The other options were early diabetes, hyperparathyroidism, and hyperthyroidism, which were declined by blood test reports. Panoramic radiography and clinical examinations led us to periodontitis as a manifestation of systematic disease, and moved the doubt toward neoplastic diseases, such as LCH which needed to be confirmed by IHC.

For this purpose, the first and second right mandibular primary teeth were anesthetized by an inferior alveolar nerve block with lidocaine 2%. There were no significant lesions; however, some surrounding soft tissues were collected for pathology assessments by a slight curettage in the socket after extraction, and then it was irrigated with normal saline 0.9%. This procedure was performed in a quite sterile and atraumatic condition to reduce possible contamination. Tissue samples were soaked in formalin and were immediately sent to the laboratory. Ibuprofen 200 mg, amoxicillin 500 mg, and metronidazole 250 mg were prescribed as analgesic and antibiotic Medications for a week. The patient was recalled 5 days after tooth extraction to evaluate the healing process and possible infection and in the next follow‐up session, extracted socket healing was normal without infection or bone necrosis (Figure 1).

**FIGURE 3:**
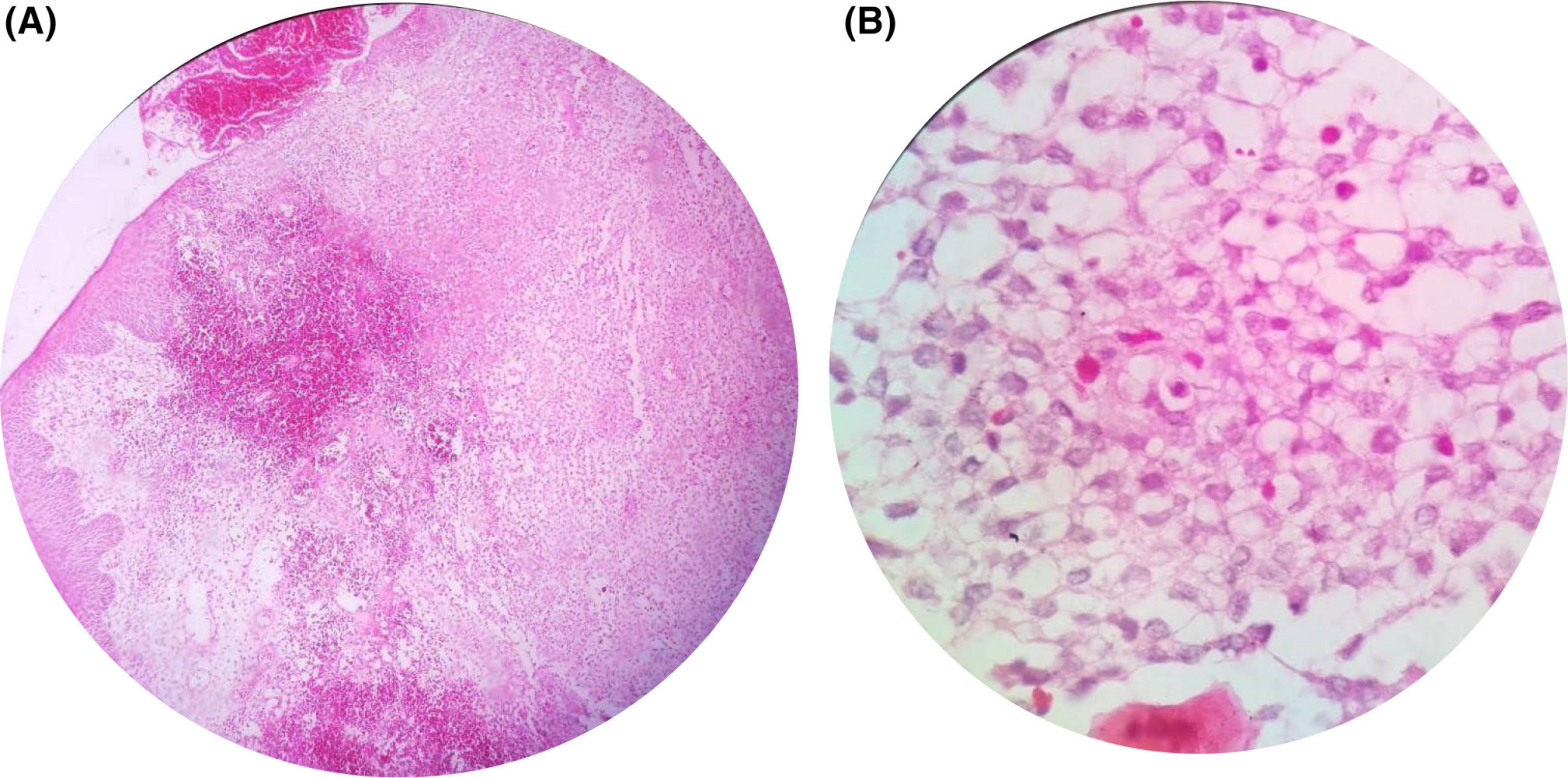
Histopathological assessment; (A) oral soft tissue segment with cell infiltration (×50), (B) hematoxylin and eosin stain (×400).

^99m^ Technetium‐methylene diphosphonate (^99m^ Tc‐MDP) skeletal scintigraphy was performed to evaluate bone involvements without sedation. The radiopharmaceutical method with ^99m^ Tc‐MDP targeted hydroxy apatite crystal in the skeleton with 6 mCi injected dose and 2.5 mSv radiation burden. The pelvic, knees, hands/feet, and cervicothoracic area were assessed. Mild osteoblastic activity in the zygomatic process of the right frontal lobe was noted. Otherwise, no remarkable active osteoblastic lesion was detected in the skeleton. One study has reported a case with LCH, indicating osteolytic lesions on zygomatic and frontal bone that led to eyelid swelling and pain.^9^ Despite the osteoblastic activities in the zygomatic process of the frontal bone in this case, no signs or symptoms were reported in this area such as eyelid swelling, pain, tenderness, or visual impairment.

IHC reported positive neoplastic cells based on S‐100 and CD1a markers, and confirmed LCH. After diagnosis, she was referred to a pediatric oncologist for treatment (Figure 3).

**FIGURE 4:**
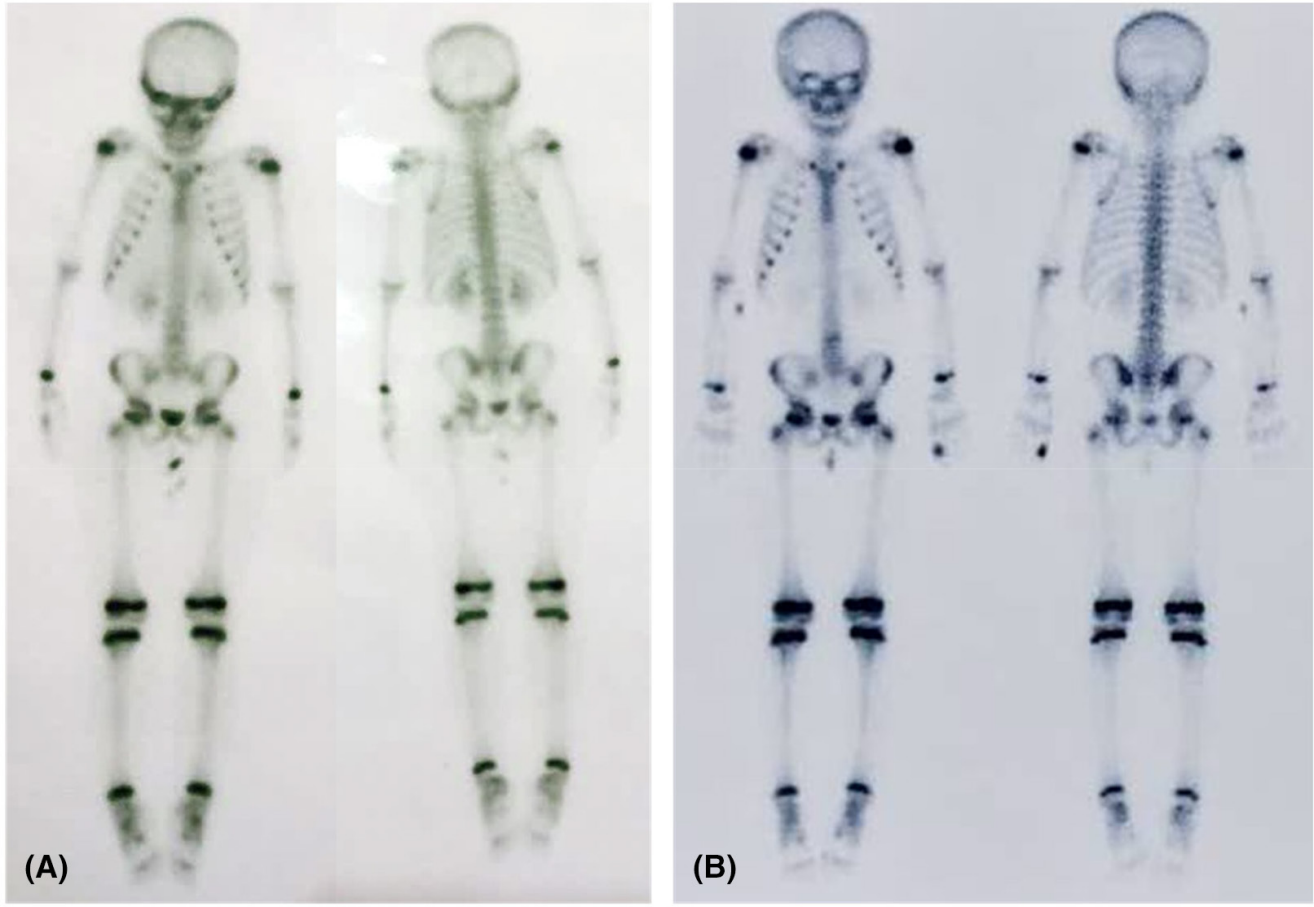
^99m^ Tc‐MDP skeletal scintigraphy; (A) before treatment. (B) seven months later, focal intense osteoblastic activity in the distal phalanx of the left‐hand second digit was seen.

The chemotherapy regimen started with vinblastine 6 mg and prednisone 5 mg. Kytril (Granisetron hydrochloride) 3 mg was injected as an antiemetic medication. Twenty days after the last chemotherapy session, a second scintigraphy was ordered with the same injected dose and radiation burden. Focal intense osteoblastic activity in the distal phalanx of the left‐hand second digit was seen along with mild osteoblastic activity in the fronto‐orbital and zygomatic process of the right orbit, indicating the progression of the disease. Right otitis media, along with elevated erythrocyte sedimentation rate (ESR), C‐reactive protein (CRP), and anemia were seen (Figure 4).

### 2.1 Diagnostic challenges

There were no significant challenges in terms of diagnosis, including financial problems or child cooperation. However, the sudden loss of their 18‐year‐old son due to an accident made the family vulnerable and sometimes resistant to follow‐up. However, it was resolved by constant recalls. See Table S1, for sum up the primary procedure for oral LCH diagnosis in a glance.

## 3 DISCUSSION

LCH is a rare condition characterized by intense proliferation and infiltration of histocytes in various tissues, either locally or generally. It primarily affects children aged one to four, males, and Caucasians, also it can occur in individuals of any age, sex, and race.^1^, ^2^ The most common sites for bone lesions are the head and neck area, particularly the skull and jaws, with a tendency to affect the mandible. Therefore, oral manifestations might be the earliest indication of this disease.^10^, ^11^ Due to nonspecific manifestations of LCH, it can be under or misdiagnosed, and delayed diagnosis can lead to life‐threatening complications. One of them is hemophagocytic lymphohistiocytosis, a fetal condition with severe hyperinflammation and lymphositis activation. Therefore dentists should be familiar with the oral manifestations.^7^, ^12^, ^13^ Clinical symptoms may include cell infiltration in the jaws, periodontium, and mucosa.^1^ Infiltration in the bone and periodontium can destroy alveolar bone and periodontal ligaments, leading to tooth hypermobility, early loss of teeth, and deep periodontal pocket during clinical examinations. In radiographic assessment, a radiolucent area with punched‐out borders may be observed. The teeth in destructed alveolar bone may appear as “floating teeth”.^1^, ^11^ Infiltration in the mucosa can cause oral ulcers, gingival enlargement, papulopustular lesions, necrotic interdental papilla, and granulomatous gingivitis.^3^, ^10^ Oral lesions may have a round or elliptic shape, erythematous color, swollen margins, and be painful to palpation^10^ (Table 1).

**TABLE 1:** Previous case reports of Langerhans cell histiocytosis (LCH) in children with oral manifestations.

Study/year	Case gender	Age	Clinical findings	Radiographical findings	Intervention
Shakya et al., 2023^22^	Male	5 Y	Gingival bleeding Gingival recession Ulcers on the hard palate Teeth mobility Pain by chewing Distortion in the fingernails Otitis media	Destored permanent mandibular folicule Interdental bone loss in molar area mild Thickening of soft tissue in hard palate in CT No skeletal metastasis in scintigraphy	Chemotherapy with VinblastineSteroid therapy with Prednisone
Meng et al., 2022^23^	Male	2 Y	Recurrent swelling of the left face Gingival necrosis Irregular ulcer Dental caries Teeth hypermobility	Periapical radiolucency Destruction of palatal plate in CBCT Osteoblastic activity in the mandible in scintigraphy	Chemotherapy with VinblastineSteroid therapy with Prednisone
Neves‐Silva et al., 2018^24^	Male	7 Y	Extensive ulcers on the left hard palate	No jawbone involvement	NM
	Male	2 Y	Lesions in hard palate mucosa and gingiva Gingival bleeding Pain Teeth mobility	Not performed	NM
	Male	2 Y	Lesions in hard palate mucosa, gingiva and alveolar ridge	Not performed	NM
Devi et al., 2015^25^	Female	4 Y	Generalized gingival erythema Bleeding on palpation Pain by chewing Generalized tooth mobility Calculus Severe halitosis	Generalized bone loss Intense osteoblastic activity in the right parietal bone of the cranium, mandible, and right femur in scintigraphy	Root scaling Short course of Metronidazole
Erdem et al., 2013^21^	Male	2 Y	Deep periodontal pockets Swelling of gingiva Gingival erythema Bleeding of gingiva around primary molars Fever Seborrheic dermatitis‐like rash History of Epstein–Barr virus infection	Deep periodontal pockets Severe bone loss of all primary molars	Chemotherapy
Mitomi et al., 2005^26^	Female	3 Y	Swelling of mucosa around primary molars Dark violet discoloration with bleeding on palpation Exophthalmos	Multiple osteolytic lesions of the skull and mandible	Chemotherapy Steroid therapy with Prednisone

Abbreviations: CBCT, cone beam computed tomography; CT, computed tomography; NM, not mentioned; Y, years old.

In addition to clinical and radiological evaluations, IHC tests must confirm the presence of CD1a and S‐100 antibodies; therefore, a biopsy of suspected tissues is required.^11^ In unifocal bone involvements, a slight curettage during biopsy may accelerate healing, and no additional intervention may be necessary.^7^ Studies have suggested that, serum alkaline phosphatase levels and thyroids hormones should be checked to explore other factors contributing to the early loss of teeth.^1^, ^10^, ^14^

In this case, periodontitis was the sole manifestation, and the patient reported neither pain nor facial swelling. The differential diagnosis considered prepubertal periodontitis due to periodontal involvement, but this was ruled out due to the absence of bacterial or hormonal factors.^15^ Radiography revealed resorption of the alveolar ridge in the area of the primary molar or permanent premolar area. The follicle of the first premolar was not intact on the right side, and the second premolar was absent. The question arises as to why the permanent premolar regions are mainly affected. The hypothesis of tooth agenesis and bone loss pattern may provide an explanation.^16^ This theory suggests that innervation plays a crucial role in alveolar bone growth and maintenance, with three main innervation patterns: (1) incisor field, (2) canine/premolar field, and (3) molar field in the maxilla or mandible. Areas further away from these nerve branches experience slower growth due to reduced innervations, including the site between the first and second premolars.^16^ This may explain the resorption of primary molar—future permanent premolar—regions in this case. In simpler terms: The further from the nerve, the more vulnerable the area becomes.

Histocyte infiltration in bone and subsequent destruction suggests that succedaneous premolars will be affected based on this hypothesis.^16^ If the pattern of destruction continues, permanent molars and premolars may be lost, necessitating a plan for the replacement of missing. Dental implants are not recommended in children with LCH, and a definitive restoration may be postponed until completion of growth in the third decade of life. In the meantime, an interim prosthesis might be beneficial if needed.^17^ For severe bone loss, free bone flaps, or microvascular‐free flaps are used for jaw reconstruction.^18^

As mentioned before, LCH is classified into single/multisystem and risk organ involvement groups.^5^ Based on the report of the first scintigraphy, mild osteoblastic activity was seen in the zygomatic process of the right frontal bone. Seven months later, second scintigraphy reported focal intense activity in the distal phalanx of the left hand in the second digit additionally. Based on the multisite bone involvement in the skull and phalanx, LCH in this patient is categorized into a multifocal risk organ group.

In addition to the physical aspect of this disease, the psychosocial problems of the patients should not be neglected. The burden of this disease is notable, especially in children with multisystem involvement. Complications, including hypothalamic–pituitary dysfunction, cognitive dysfunction, and cerebellar involvement, were detected in survivors.^19^ More internalizing problem behaviors were seen in children with LCH, and even they remained dependent in their adult life.^19^, ^20^

In this patient with LCH, no cutaneous lesions or systematic involvements were seen; contrary to other studies.^21^, ^22^ Oral manifestation, which was solely an idiopathic attachment loss, was present. Dentists must be aware of possible signs and symptoms for patient screening. Any uncommon attachment loss, gingival recession, and early pathologic tooth mobility with no significant calculus or other causes should be promptly considered for a biopsy for further evaluation. The cooperation of dentists, oncologists, dermatologists, endocrinologists, and even psychologists is beneficial in diagnosing and managing LCH.

## 4 CONCLUSION AND TAKEAWAY LESSON

This article demonstrated the critical role of dentists' subtle diagnosis of LCH. Any pathologic mobility that does not match the normal age of tooth shedding must be evaluated for further assessment. It should be noted that early detection and treatment of LCH increase the survival rate and quality of future life among children dramatically.

## AUTHOR CONTRIBUTIONS

**Bahareh Nazemisalman:** Conceptualization; data curation; formal analysis; funding acquisition; investigation; methodology; project administration; resources; software; supervision; validation; visualization; writing – original draft; writing – review and editing. **Mobina Sadat Zarabadi:** Conceptualization; data curation; formal analysis; funding acquisition; investigation; methodology; project administration; resources; software; supervision; validation; visualization; writing – original draft; writing – review and editing.

## FUNDING INFORMATION

There were no specific grants or funding for this article.

## CONFLICT OF INTEREST STATEMENT

The authors declare that they have no conflict of interest.

## CONSENT

Written informed consent was taken from the child's guardians, which were her parents. This consent included using laboratory tests, imaging, demographic data, and the treatment process.

## Supporting information


Data S1.

## Data Availability

The data used in this article is available upon request from the authors.
