# Supplementary material for: Oral manifestation as the only sign of Langerhans cell histiocytosis: A case report
Source: Clin Case Rep. 2024 Jan 10;12(1):e8410. doi: 10.1002/ccr3.8410 (PMC10781892; doi:10.1002/ccr3.8410)
Supplement: Supplementary file 1 — Data S1. [file CCR3-12-e8410-s001.docx]

| **1) Clinical examination** | **2) Oral examination** | **3) Blood tests** | **4) Imaging** | **5) Histopathology** |
| --- | --- | --- | --- | --- |
| - Ophthalmological - Dermatological - Cardiac - Neurological - Endocrinal - Oral - Gastrointestinal | - Attachment loss - Deep pocket depth - Bleeding on probing - Gingival inflammation - Gingival recession - Tooth mobility - Calculus - Oral pain - Halitosis - Tooth caries - Tenderness to palpation | - WBC diff. - RBC - Hgb - HCT - MCV/MCH/MCHC - Platelets - ESR - FBS - Thyroid hormones - Alkaline phosphatase - ALT - AST - INR / PT/PTT | - Panoramic - Chest radiography - Skull radiography - Abdominal ultrasound(spleen or liver) - Scintigraphy | - IHC for S-100 and CD1a - Histologic analysis |

**Supplementary Table 1.** **The primary procedure for oral LCH diagnosis in a glance.** Each column demonstrated the clinical and paraclinical procedure for LCH diagnosis step by step.
